# Branched-chain amino acids supplementation improves liver frailty index in frail compensated cirrhotic patients: a randomized controlled trial

**DOI:** 10.1186/s12876-023-02789-1

**Published:** 2023-05-15

**Authors:** Sith Siramolpiwat, Nisakorn Limthanetkul, Bubpha Pornthisarn, Ratha-korn Vilaichone, Soonthorn Chonprasertsuk, Patommatat Bhanthumkomol, Pongjarat Nunanan, Navapan Issariyakulkarn

**Affiliations:** 1 Chulabhorn International College of Medicine, Thammasat University, Pathum Thani, Thailand; 2 Division of Gastroenterology, Department of Internal Medicine, Faculty of Medicine, Thammasat University, Pathum Thani, Thailand

**Keywords:** Cirrhosis, Chronic liver disease, Frailty, Malnutrition, Sarcopenia

## Abstract

**Background:**

Physical frailty is related with morbidity and mortality in patients with cirrhosis. Currently, there is no approved treatment of frailty in these patients. Here, we evaluated the efficacy of 16 weeks branched-chain amino acids (BCAA) supplementation on frailty in frail compensated cirrhotic patients.

**Methods:**

After a 4-week run-in period consisted of dietary and exercise counseling, compensated cirrhotic patients with frailty, defined by liver frailty index (LFI)≥4.5, were randomly assigned (1:1) to BCAA or control group. The BCAA group received twice daily BCAAs supplementation (210 kcal, protein 13.5 g, BCAA 2.03 g) for 16 weeks. The primary outcome was frailty reversion. The secondary outcomes were changes in biochemistries, body composition evaluated by bioelectrical impedance analysis, and quality of life (QoL).

**Results:**

54 patients were prospectively enrolled (age 65.5 ± 9.9 years, 51.9% female, Child-Pugh A/B 68.5%/31.5%, MELD 10.3 ± 3.1). Baseline characteristics were similar between both groups. At week 16, BCAA group had a significant improvement in LFI (-0.36 ± 0.3 vs. -0.15 ± 0.28, P = 0.01), BMI (+ 0.51 ± 1.19 vs. -0.49 ± 1.89 kg/m^2^, P = 0.03), and serum albumin (+ 0.26 ± 0.27 vs. +0.06 ± 0.3 g/dl, P = 0.01). The proportion of frailty reversion at week 16 was significantly higher in BCAA group (36% vs. 0%, P < 0.001). Compared with baseline, BCAA group had a significant increase in skeletal muscle index (7.5 ± 1.6 to 7.8 ± 1.5 kg/m^2^, P = 0.03). Regarding the QoL, only the BCAA group had a significant improvement in all 4 domains of physical component score of the SF-36 questionnaire.

**Conclusions:**

A 16-week BCAA supplementation improved frailty in frail compensated cirrhotic patients. In addition, this intervention resulted in an improvement of muscle mass and physical domain of QoL in these patients.

**Trial registration:**

This study was registered with Thai Clinical Trial Registry (TCTR20210928001; https://www.thaiclinicaltrials.org/#).

## Introduction

Frailty is a complex syndrome characterized by a decrease in physiologic reserve and increased vulnerability to health stressors. In patients with cirrhosis, frailty is commonly reported as decreased physical function, decreased physical performance, and disability [1, 2]. The reported prevalence of frailty in patients with cirrhosis ranges from 18 to 43% [3, 4]. Regarding the clinical impact, frailty has been shown to be linked with worsened clinical outcomes in patients with decompensated cirrhosis; for example, waitlist mortality, hospitalization, and further decompensations [5–7]. In compensated cirrhosis, previous study has demonstrated that frailty was significantly associated with developing new decompensations and unplanned hospitalization [8]. In addition, frailty is associated with the development of falls, depression, disability, and impaired quality of life (QoL) [3, 8, 9]. Notably, an improvement of frailty scores over time has been shown to be associated with a better clinical outcome [10]. Therefore, therapeutic interventions targeting at frailty reversion in cirrhosis is urgently needed.

Branched-chain amino acids (BCAA) including leucine, isoleucine and valine are responsible for ammonia detoxification and protein synthesis. In patients with cirrhosis, BCAA also serve a preferential source of energy for skeletal muscle [11]. A decrease in plasma BCAA, commonly found in cirrhosis, is caused by several factors; for example, hyperammonemia, chronic inflammation, hormonal changes, and starvation. Indeed, a low serum Fischer’s ratio (BCAA to aromatic amino acids ratio) has been shown to be associated with a worse prognosis in patients with cirrhosis [12]. To date, current guidelines do not support routinely supplementation with BCAA in all patients with cirrhosis given that the clinical trials evaluating the role of BCAA in cirrhotic-related complications yielded conflicting results [1, 13]. However, some studies have demonstrated a decrease in clinical events and improved QoL with oral BCAA supplementation in cirrhotic patients [14].

So far, the role of BCAA supplementation on muscle improvement in patients with cirrhosis has not been well determined. Previous studies assessing the role of oral BCAA supplementation on frailty and/or sarcopenia in cirrhosis are limited and demonstrated inconsistent results [15–17]. We, therefore, conducted this open-labeled randomized controlled trial to evaluate the role of oral BCAA supplementation for 16 weeks on frailty reversion in patients with compensated cirrhosis.

## Materials and methods

### Trial design

The present study was an investigator-initiated, single center, parallel, open-labeled randomized controlled trial conducted in Thammasat University Hospital, Thailand, from July 2021 to June 2022. This study was approved by the Institutional Review Board of Thammasat University and was retrospectively registered with Thai Clinical Trial Registry number TCTR20210928001 (first posted date: 28/09/2021). The study protocol conforms to the ethical guidelines of the 1975 Declaration of Helsinki.

### Patients

All patients diagnosed with frail stable Child-Pugh A or B cirrhosis aged between 18 and 75 years were eligible for inclusion in this study. The diagnosis of cirrhosis was based on a combination of clinical, biochemical, and radiographic data or histology if available. Frailty was diagnosed based on the Liver Frailty Index (LFI) ≥ 4.5. The exclusion criteria were as followed: current or recent (within the previous 6 months) clinical signs or symptoms of hepatic decompensation (grade 2 or 3 ascites, portal hypertension (PHT)-related gastrointestinal (GI) bleeding, hepatic encephalopathy (HE), or acute kidney injury (AKI)), history of hospitalization within the previous 6 months, presence of hepatocellular carcinoma (HCC) or other malignancies, received BCAA supplementation within 1 year, severe chronic heart or pulmonary diseases, chronic hemodialysis, pregnancy, active alcohol intake, poorly-controlled diabetes (hemoglobin A1C > 7%), patients who cannot follow commands or answer the questionnaire, patients who experienced recent joint pain, vertigo, dizziness, or functional disabilities during the inclusion period, and refusal to participate in this study.

### Frailty assessment

Frailty was assessed by LFI, which consists of the following 3 performance-based tests: (1) Grip strength: the average of 3 trials, measured in the subject’s dominant hand using a hand dynamometer (CAMRY digital hand dynamometer EH101); (2) Timed chair stands: measured as the number of seconds it takes to do 5 chair stands with the subject’s arms folded across the chest; and (3) Balance testing: measured as the number of seconds that the subject can balance in 3 positions (feet placed side-to-side, semi-tandem, and tandem) for a maximum of 10 s each [5]. With these 3 individual tests, the LFI was calculated using the following equation (calculator available at: http://liverfrailtyindex.ucsf.edu): (− 0.330×gender-adjusted grip strength)+(− 2.529×number of chair stands per second)+(− 0.040×balance time) + 6. The classifications of frailty were determined by using the previously established cutoffs with robust defined as LFI < 3.2, pre-frail as LFI between 3.2 and 4.4, and frail as LFI ≥ 4.5.

### Study protocol

#### Baseline assessment and run-in period

In patients who agreed to participate, demographic data, etiologies of cirrhosis, underlying diseases, and standard laboratory parameters, including complete blood count, blood urea nitrogen (BUN), creatinine, and liver function test were recorded. The Child-Pugh score, MELD, and MELD-Na scores were calculated to assess the severity of cirrhosis. Body composition was determined by bioelectrical impedance analysis (BIA) (HBF-514c, Omron, Japan) to measure body weight, muscle mass, and fat-free mass. The skeletal muscle index (SMI) was measured as the muscle mass of all four limbs divided by height squared.

After the baseline assessment, all eligible patients underwent a 4-week run-in period, which consisted of dietary and exercise counseling. Trained dieticians provided nutritional guidance to each patient, considering a target calorie intake of 25–30 kcal/day along with 1-1.2 g/kg/day of protein intake. Regarding exercise counseling, a home-based exercise of approximately 30–45 min/day to achieve a target of 150 min/week was recommended to all patients. At the week 2 of run-in period, all patients were followed-up by phone call to ensure their adherence to dietary and exercise advice.

#### Study intervention

After the run-in period, patients were randomly assigned in a 1:1 ratio to BCAA or control group. The randomization sequence was generated by computer in block of four and stratified to Child-Pugh A or B. In the BCAA group, patients received 16-week of BCAA supplementation (Aminolaban-oral, Otsuka Pharmaceutical Co. Ltd, Thailand) twice daily. Amoinolaban-oral was orange flavor powder, and patients were advised to dissolve 5 scoops in 180 ml of water and drink in the morning and between 7 and 9 pm. Each serving (50 g, 210 kcal) contains 13.5 g of protein (BCAA: 2.03 g of leucine, 1.76 g of isoleucine, and 1.635 g of valine), 32.4 g of carbohydrate, and 3.5 g of fat. Empty containers were kept and returned at each visit to accurately assess compliance, ≥ 90% adherence was considered adequate to continue with the eligibility. In the control group, there were advised not to take BCAA or any nutritional supplementation during the study period. All patients were continued on standard medical treatment for etiology-specific and complications of cirrhosis. A telephone visit was made every 2 weeks by the study investigators to ensure the patients’ adherence to the study protocol. All patients were followed at the outpatient clinic at week 8 and 16. Clinical and biochemical data, including LFI and body composition were collected at each visit. Throughout the study period, LFI was assessed by the study personnel who were unaware of the treatment arm to reduce possible biases. In addition, participants were asked to record their nutritional intake timing and amount in the paper log provided by the investigators.

#### Study outcomes

The primary outcome of the present study was to compare the proportion of patients with frailty reversion, which was defined by the LFI < 4.5, between BCAA and control groups at week 16. The secondary outcomes included changes in LFI, body composition, laboratory parameters at week 8 and 16, and changes in QoL at week 16. QoL was assesses at baseline and at week 16 by the Thai version of the SF-36 questionnaire. This translated version of the SF-36 has been previously validated [18]. The SF-36 questionnaire consists of 36 multiple-choice questions, split into four domains in the area of physical health (physical functioning, role limitation-physical, bodily pain, general health), and four in the area of mental health (role limitation-emotional, vitality, mental health, and social functioning). Two comprehensive indexes of health-related quality of life (HRQoL) were computed: the physical component score and the mental component score. Possible scores range from 0 to 100, and a lower score indicates poorer health status.

### Statistical analysis

As there was no previous study specifically evaluated the role of BCAA on frailty on cirrhosis, the sample size was calculated based on the benefit of BCAA in cirrhotic patients with sarcopenia [15, 17]. We hypothesized that BCAA supplementation could reverse frailty by 40%. Using a two-tailed test, 24 patients were required in each group, for a *P* value < 0.05 with an alpha error of 5% and a beta error of 10%. Assuming a dropout rate of 10%, the study size should be 27 patients in each group.

The results were expressed as mean ± standard deviation (SD) or median with range. Comparisons within and between groups were performed using paired and unpaired Student’s *t*-test, respectively. For categorical data, Chi-square test or Fisher’s exact test was applied. Due to small sample size, the absolute changes (delta) in clinical and laboratory parameters between 2 groups were compared by non-parametric Mann–Whitney U tests. In addition, repeated measures ANOVA analysis was used to compare the effect of intervention over the follow-time by using the outcomes as the dependent variable and time, study group, and time-by-group interaction as the independent variables. A value of P < 0.05 was taken as statistically significant. All data were analyzed by using STATA version 13.0 (Stata Corp, Texas, USA).

## Results

### Patients

**Fig. 1:**
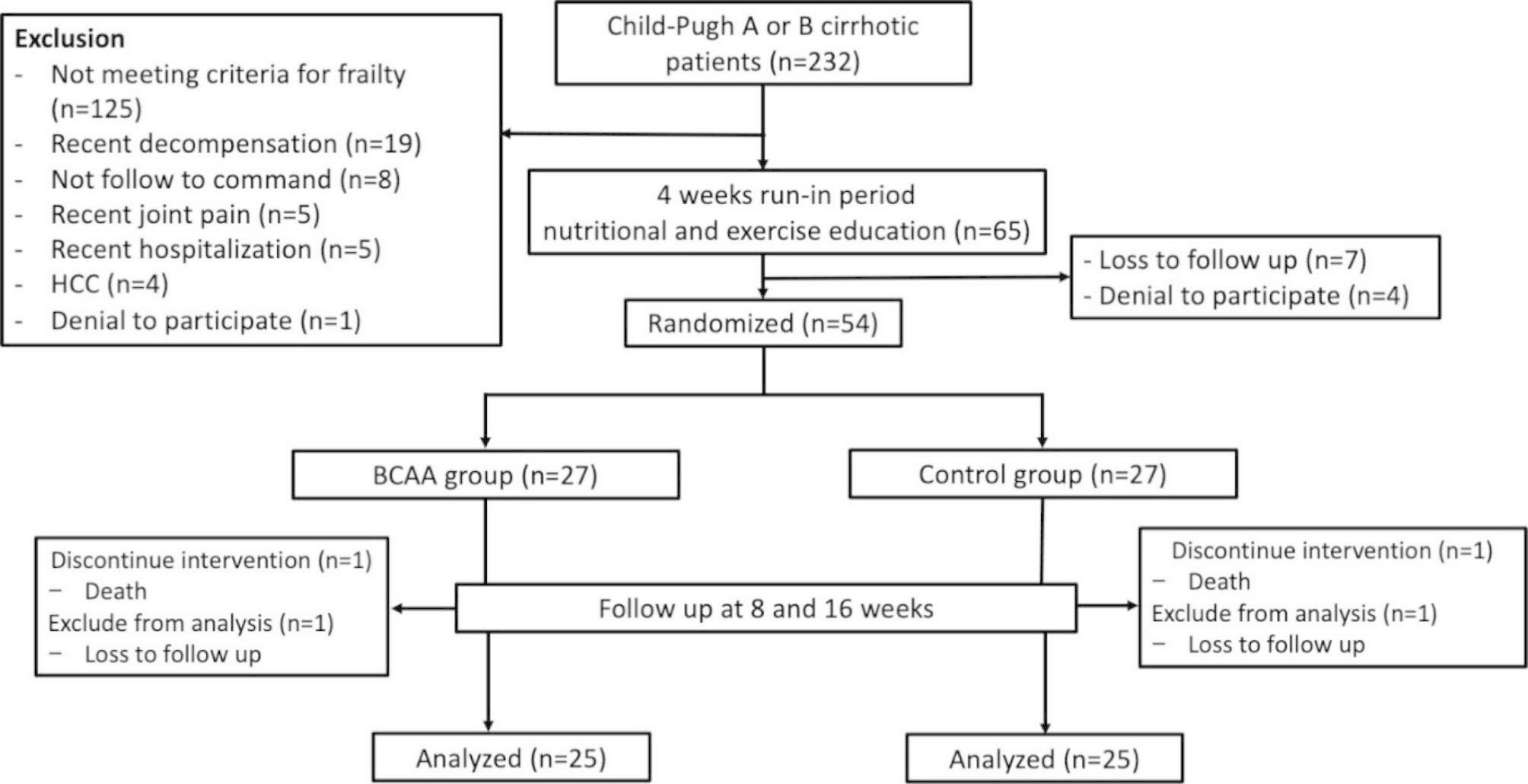
A flowchart of patients entered into the study and included in the analysis (consort diagram)

From January 2021 to March 2022, a total of 232 outpatients with Child-Pugh A or B cirrhosis were evaluated for inclusion in the study, of which 125 patients were excluded because they did not meet the LFI criteria for frailty. Thirty-eight additional patients were excluded for other reasons outlined in the Fig. 1 (consort diagram). Therefore, 65 patients participated in the run-in period, and 11 patients were subsequently excluded (7 were lost to follow up, and 4 denied to further participate in the study). Finally, a total of 54 patients were randomly assigned to BCAA group (n = 27) or control group (n = 27). Baseline characteristics of participants are shown in the Table 1. The mean age was 65.5 ± 9.9 years, and 28 patients (51.9%) were female. The main etiologies of cirrhosis were chronic hepatitis B virus infection (38.9%), alcoholism (25.9%), and chronic hepatitis C virus infection (14.8%). All patients with alcoholic cirrhosis were abstinent from alcohol drinking for > 6 months. In viral-associated cirrhosis, all patients had negative or stable viral load tests at the study inclusion; therefore, none of them required changes or initiation of anti-viral medications during the whole study period. Regarding the cirrhosis severity, 37 patients (68.5%) were Child-Pugh A and 17 patients (31.5%) were Child-Pugh B. The mean MELD and MELD-Na scores were 10.3 ± 3.1 and 12.6 ± 4.1, respectively. Seventeen patients (31.5%) had a previous history of hepatic decompensation (PHT-related GI bleeding in 13, ascites in 3, and HE in 1). The mean duration between previous decompensation events and study enrollment was 15.1 ± 3.1 months.

**Table 1:** Baseline characteristics of all included patients and by treatment group

Patient’s characteristic	Total (N = 54)	BCAA group (N = 27)	Control group (N = 27)	P value*
Age (years)	65.5 ± 9.9	64.9 ± 10.3	66.1 ± 9.6	0.64
Female sex, n(%)	28 (51.9%)	14 (51.9%)	14 (51.9%)	1.00
Weight (kg)	65.9 ± 12.9	63.6 ± 13.6	68.2 ± 12.2	0.18
Height (cm)	160.3 ± 8.4	159.1 ± 8.2	161.6 ± 8.5	0.27
BMI (kg/m^2^)	25.7 ± 4.8	24.9 ± 4.7	26.5 ± 5.0	0.26
Causes of cirrhosis, n(%)				
- HBV	21 (38.9%)	9 (33.4%)	12 (44.5%)	0.62
- Alcohol	14 (25.9%)	8 (29.6%)	6 (22.2%)	
- HCV	8 (14.8%)	4 (14.8%)	4 (14.8%)	
-Non-alcoholic fatty liver disease	4 (7.4%)	2 (7.4%)	2 (7.4%)	
- Others	7 (13.0%)	4 (14.8%)	3 (11.1%)	
Child-Pugh class, n(%)				
- A	37 (68.5%)	18 (667%)	19 (66.7%)	1.00
- B	17 (31.5%)	9 (33.3%)	8 (29.6%)	
MELD score	10.3 ± 3.1	10.4 ± 3.5	10.3 ± 2.8	0.86
MELD-Na score	12.6 ± 4.1	12.5 ± 4.5	12.7 ± 3.7	0.84
Laboratory parameters				
- Hb (g/dl)	12.2 ± 1.4	12.4 ± 1.5	11.9 ± 1.4	0.16
- WBC (x10^3^/ul)	5.3 ± 1.8	5.5 ± 2.0	5.1 ± 1.7	0.49
- Platelet (x10^3^/ul)	138.9 ± 74.6	131.7 ± 63.9	145 ± 84.7	0.52
- BUN (mg/dl)	13.5 ± 4.0	13.1 ± 3.9	14 ± 4.2	0.39
- Creatinine (mg/dl)	0.9 ± 0.3	0.8 ± 0.3	1.0 ± 0.3	0.07
- AST (U/L)	45.1 ± 19.2	47.9 ± 22.9	42.3 ± 14.5	0.28
- ALT (U/L)	28.8 ± 14.4	29.9 ± 25.2	27.7 ± 13.7	0.56
- ALP (U/L)	98.2 ± 38.4	104.4 ± 44.4	91.9 ± 30.1	0.23
- Total protein (g/dl)	7.5 ± 0.5	7.6 ± 0.6	7.4 ± 0.4	0.17
- Albumin (g/dl)	3.5 ± 0.6	3.4 ± 0.6	3.5 ± 0.5	0.56
- Total bilirubin (mg/dl)	1.4 ± 1	1.6 ± 1.2	1.2 ± 0.69	0.11
- aPTT (sec)	27.5 ± 4.9	26. ±4.1	28.3 ± 5.7	0.27
- PT (sec)	14.0 ± 2.1	13.9 ± 2.0	14.3 ± 2.1	0.87
- HbA1C (%)	5.5 ± 0.5	5.4 ± 0.5	5.6 ± 0.6	0.35
LFI	5.1 ± 0.4	5.0 ± 0.4	5.2 ± 0.5	0.14
Body composition				
- Muscle mass (kg)	19.7 ± 4.7	19.3 ± 5.4	20.1 ± 3.8	0.52
- SMI (kg/m^2^)	7.7 ± 1.4	7.5 ± 1.6	7.8 ± 1.2	0.58
- Fat free mass (kg)	45.9 ± 8.5	45.2 ± 9.9	46.5 ± 7.1	0.59

^*^P-value for comparison between BCAA vs. control group

BMI, body mass index; HBV, hepatitis B virus; HCV, hepatitis C virus; MELD, model for end-stage liver disease; Hb, hemoglobin; WBC, white blood cell; BUN, blood urea nitrogen; AST, aspartate transaminase; ALT, alanine transaminase; ALP, alkaline phosphatase; aPTT, activated partial thromboplastin time; PT, prothrombin time; LFI, liver frailty index; SKI, skeletal muscle index

As shown in the Table 1, there was no significant difference in clinical and laboratory parameters between the two groups at the time of enrollment. The mean LFI was 5.0 ± 0.4 and 5.2 ± 0.5 in the BCAA and control group, respectively. During follow-up, 2 patients dropped out in each group. In the BCAA group, 1 patient died from hemorrhagic stroke at 32 days, and 1 patient was lost to follow-up at 60 days after randomization. In the control group, 1 patient died from urinary tract infection with sepsis at 31 days, and 1 patient was lost to follow-up at 92 days after randomization. Finally, total of 50 patients completed the 16-week study protocol.

### Changes in parameter over the follow-up period

Table 2 shows changes in clinical and laboratory parameters from baseline to week 8 and 16 within each group. As shown, there was a significant increase in BMI and serum albumin at week 16 in the BCAA group (24.9 ± 4.7 to 25.5 ± 4.9 kg/m^2^, P = 0.004, and 3.5 ± 0.6 to 3.8 ± 0.6 g/dl, P < 0.001, respectively). Whereas there was no significant changes in both BMI and serum albumin in the control group. In terms of cirrhosis severity, there was no significant change in the Child-Pugh, MELD and MELD-Na scores between baseline and at week 16 in both groups. Regarding the frailty assessment, there was a significant improvement in the LFI at week 16 in both groups (BCAA, 5.0 ± 0.4 to 4.7 ± 0.4, P < 0.001; control, 5.2 ± 0.5 to 5.0 ± 0.5, P = 0.01). However, only the BCAA group had a significant improvement in all 3 frailty tests; meanwhile, the control group had significant improvement in only the hand grip strength test. In addition, there was a significant increase in SMI at week 16 in the BCAA group (7.5 ± 1.6 to 7.8 ± 1.5 kg/m^2^, P = 0.03). Whereas the SMI in the control group remained unchanged, and there were no significant changes in the fat free mass in both groups.

**Table 2:** Changes in clinical and laboratory parameters from baseline to week 8 and 16 within each group

	BCAA group					Control group				
	Baseline	At 8 weeks	P-value*	At 16 weeks	P-value**	Baseline	At 8 weeks	P-value*	At 16 weeks	P-value**
BMI (kg/m^2^)	24.9 ± 4.7	25.1 ± 5.0	0.38	25.5 ± 4.9	0.04	26.5 ± 5.0	26.5 ± 5.1	0.83	26.1 ± 4.7	0.21
Serum albumin (g/dl)	3.5 ± 0.6	3.6 ± 0.6	0.02	3.8 ± 0.6	< 0.001	3.5 ± 0.5	3.6 ± 0.6	0.65	3.6 ± 0.5	0.36
Child-Pugh score	6.3 ± 1.3	6.1 ± 1.4	0.12	5.9 ± 1.2	0.058	5.9 ± 1.3	6.0 ± 1.2	0.38	5.9 ± 1.4	0.71
MELD	10.4 ± 3.5	10.0 ± 3.0	1	9.4 ± 2.6	0.33	10.3 ± 2.8	10.0 ± 3.1	0.48	10.1 ± 3.5	0.94
MELD-Na	12.5 ± 4.5	11.5 ± 3.5	0.5	10.7 ± 3.1	0.07	12.7 ± 3.7	11.9 ± 3.9	0.26	11.6 ± 4.0	0.28
LFI	5.0 ± 0.4	4.8 ± 0.4	0.02	4.7 ± 0.4	< 0.001	5.2 ± 0.5	5.0 ± 0.5	0.03	5.0 ± 0.5	0.01
HGS (kg)	15.4 ± 5.9	21.1 ± 8.2	< 0.001	22.0 ± 8.2	< 0.001	13.2 ± 4.7	18.6 ± 7.3	< 0.001	18.8 ± 5.5	< 0.001
CST (second)	35.6 ± 10.1	29.8 ± 10.5	0.01	26.3 ± 10.1	< 0.001	34.5 ± 9.9	34.1 ± 10.2	0.52	32.5 ± 10.21	0.02
Balancing test (second)	24.8 ± 3.9	26.3 ± 3.0	0.02	28.3 ± 2.8	< 0.001	23.2 ± 5.5	24.0 ± 6.6	0.26	24.6 ± 6.5	0.07
Body composition										
- Muscle mass (kg) - SMI (kg/m^2^) - Fat free mass (kg)	19.3 ± 5.4 7.5 ± 1.6 45.2 ± 9.9	19.6 ± 5.3 7.7 ± 1.6 44.9 ± 9.8	0.26 0.26 0.52	19.9.2 ± 5.4 7.8 ± 1.5 45.1 ± 10.4	0.06 0.03 0.76	20.1 ± 3.8 7.8 ± 1.2 46.5 ± 7.1	20.2 ± 3.4 7.8 ± 1.1 46.9 ± 6.5	0.92 0.90 0.44	20.2.2 ± 3.6 7.8 ± 1.1 46.6 ± 6.7	0.95 0.97 0.96

*P-value for comparison between baseline vs. week 8

**P-value for comparison between baseline vs. week 16

BMI, body mass index; MELD, model for end-stage liver disease; LFI, liver frailty index; HGS, hand grip strength; CST, chair stands test; SKI, skeletal muscle index

### Changes in parameters between the two groups

Comparisons of changes (delta, Δ) in clinical and laboratory parameters at baseline and week 16 between 2 groups is demonstrated in the Table 3. Compared with the control group, those who were randomized to BCAA had a significant increase serum albumin (Δ albumin, + 0.26 ± 0.27 vs. +0.06 ± 0.3 g/dl, P = 0.02). However, there was no significant difference in the changes in Child-Pugh, MELD, and MELD-Na scores between 2 groups. Regarding the frailty assessment, there was a significant reduction in LFI in the BCAA group compared with the control group (Δ LFI, -0.36 ± 0.3 vs. -0.15 ± 0.28, P = 0.01). Considering each frailty assessment individually, BCAA group had a significant improvement in the chair stand test (Δ CST, -9.28 ± 11.05 vs. -2 ± 4.04 s, P < 0.01), and balancing test (Δ BT, 3.52 ± 3.18 vs. 1.32 ± 3.49 s, P = 0.02). However, there was no significant difference in the change in hand grip strength between both groups.

**Table 3:** Comparison of changes in parameters from baseline to week 16 between 2 groups

Delta (baseline and week 16)	BCAA group	Control group	P-value^*^
∆ BMI (kg/M^2^)	0.51 ± 1.19	-0.49 ± 1.89	0.05
∆ Albumin (g/dL)	0.26 ± 0.27	0.06 ± 0.3	0.02
∆ Child-Pugh score	-0.36 ± 0.9	0.08 ± 1.08	0.46
∆ MELD score	-0.4 ± 2.02	-0.04 ± 2.46	0.67
∆ MELD-Na score	-0.64 ± 1.73	-0.68 ± 3.1	0.61
∆ LFI - ∆ HGS (kg) - ∆ CST (sec) - ∆ Balancing test (sec)	-0.36 ± 0.3 6.63 ± 3.77 -9.28 ± 11.05 3.52 ± 3.18	-0.15 ± 0.28 5.69 ± 3.13 -2 ± 4.04 1.32 ± 3.49	0.01 0.52 < 0.01 0.02
Body composition			
- ∆ Muscle mass (kg) - ∆ SMI (kg/m^2^) - ∆ Fat free mass (kg)	0.52 ± 1.31 0.21 ± 0.52 -0.15 ± 2.36	-0.03 ± 2.56 -0.01 ± 1.04 0.40 ± 4.06	0.08 0.07 0.85

*By non-parametric Mann–Whitney U test

BMI, body mass index; MELD, model for end-stage liver disease; LFI, liver frailty index; HGS, hand grip strength; CST, chair stands test; SKI, skeletal muscle index

**Fig. 2:**
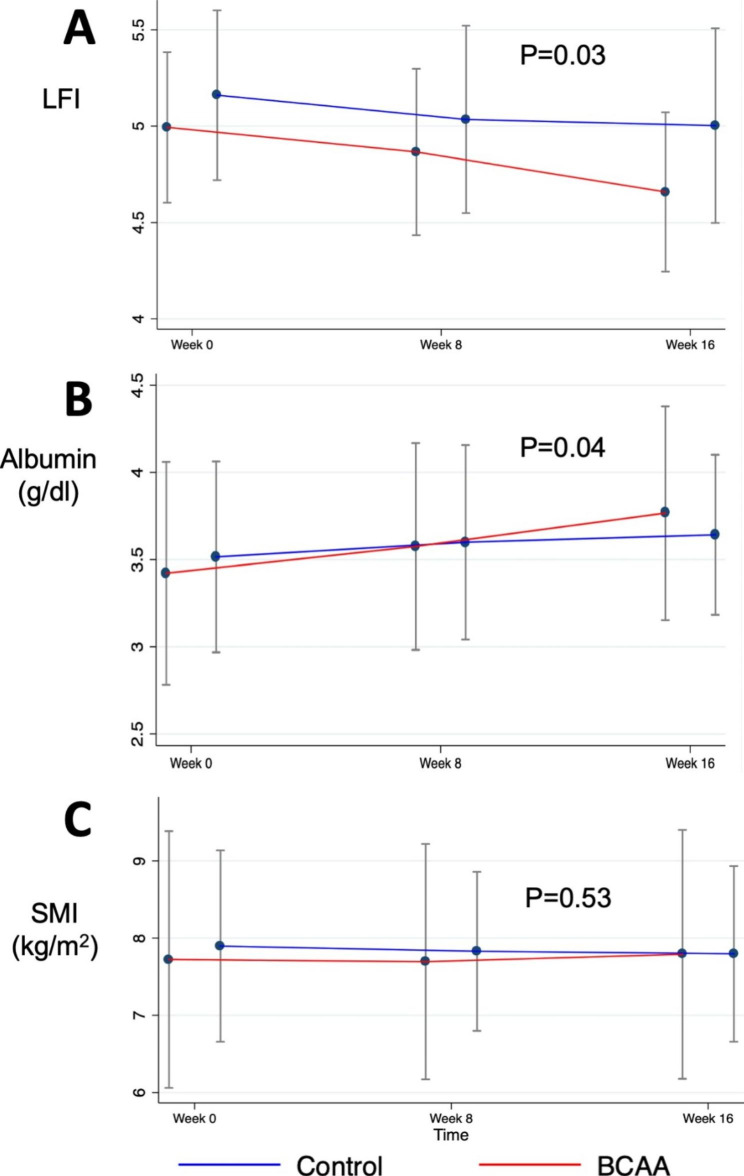
Comparison of changes of liver frailty index (Fig. 2A), serum albumin (Fig. 2B), and skeletal muscle index (Fig. 2C) over the 16-week follow-up period between 2 groups

By repeated measures ANOVA analysis using each outcome as the dependent variable and time, study group, and time-by-group interaction as the independent variables, BCAA group had a significant increase in LFI (P = 0.03), and serum albumin (P = 0.04), but not SMI (P = 0.53). Figure 2 shows changes in these 3 parameters over the 16-week follow-up period.

### Reversion of frailty

At week 8, there was a trend toward higher proportion of frailty reversion in the BCAA group than in the control group (15.4% vs. 0%, P = 0.06). At week 16, the proportion of frailty reversion was significantly higher in the BCAA group (36% vs. 0%, P < 0.001). Subgroup analysis was done separately in Child-Pugh A and B cirrhosis. The proportion of frailty reversion at week 16 was significantly higher in the BCAA group in both Child-Pugh A and B patients (Child A, 27.8% vs. 0%, P = 0.02, and Child B, 57.1% vs. 0%, P = 0.049).

### Changes in quality of life at week 16

At week 16, the BCAA group had a significant improvement in all 4 domains of physical component score (physical function, role physical, bodily pain, and general health) (Table 4). Of note, the physical component score was significantly increased at week 16 in the BCAA group (44.1 ± 8.0 at baseline to 49.1 ± 6.9 at week 16, p < 0.001). Meanwhile, there were no significant changes in the physical component score as well as all its 4 subscores in the control group. There were no significant changes in the mental component score as well as its 4 subscores in both groups.

**Table 4:** Changes in quality of life as evaluated by the SF-36 questionnaire from baseline to week 16 within each group

SF-36 questionnaire (mean ± SD)	BCAA group			Control group		
	Baseline	At 16 weeks	P-value	Baseline	At 16 weeks	P-value
Physical function	61.0 **±** 25.3	73.8 **±** 21.4	< 0.001	65.0 **±** 23.8	65.2 **±** 21.9	0.9
Role physical	73.0 **±** 27.9	88.0 **±** 17.9	0.006	66.0 **±** 26.9	61.0 **±** 30.7	0.096
Bodily pain	73.9 **±** 20.2	82.6 **±** 14.1	0.02	74.4 **±** 16.9	75.1 **±** 15.8	0.66
General health	55.8 **±** 19.6	65.0 **±** 16.9	0.02	52.4 **±** 17.4	54.0 **±** 18.3	0.43
**Physical component score**	**44.1 ± 8.0**	**49.1 ± 6.9**	**< 0.001**	**44 ± 8**	**43.1 ± 8.2**	**0.08**
Role emotion	81.3 **±** 23.7	90.7 **±** 15.3	0.09	74.7 **±** 24.1	78.7 **±** 23.3	0.33
Vitality	68.0 **±** 17.7	74.6 **±** 13.1	0.03	68.0 **±** 16.4	69.8 **±** 14.5	0.29
Mental health	82.1 **±** 13.6	85.3 **±** 10.3	0.18	81.9 **±** 11.4	83.8 **±** 9.8	0.13
Social functioning	75.0 **±** 19.4	80.0 **±** 17.7	0.08	77.0 **±** 15.6	80.5 **±** 14.9	0.07
**Mental component score**	**55.2 ± 6.2**	**56.7 ± 4.5**	**0.2**	**53.7 ± 5.6**	**54.9 ± 5.6**	**0.07**

### Adverse events and patient compliance

There was no patient experienced major adverse event during the study. One patient temporarily discontinued BCAA for 1 week during hospital admission because of non-PHT related upper gastrointestinal bleeding at week 9 of the study. The rest of participants reported > 90% compliance to the study medications. None was documented with a new episode of hepatic decompensation during the study.

## Discussion

The main result of this open-label randomized controlled trial was that a 16-week BCAA supplementation was associated with an improvement of LFI in frail Child-Pugh A and B cirrhotic patients. Of note, BCAA treatment resulted in a 36% frailty reversion rate compared to 0% in the control group. Moreover, the administration of BCAA also increased muscle mass and improved the physical component of QoL in these patients.

Frailty, a distinct biologic syndrome of decreasing physiologic reserve and increase vulnerability to health stressors, is currently known as a poor prognostic factor in patients with cirrhosis [4–7, 19]. It has been shown to be related with decompensation events and death in cirrhotic patients listed for liver transplantation [20]. In addition, frailty is associated with an increased risk of unplanned hospitalization and hepatic decompensation in patients with compensated cirrhosis [8]. In terms of QoL, presence of frailty significantly decreased both physical and mental component scores of the SF-36 questionnaire. Notably, a recent multicenter study in patients with compensated and decompensated cirrhosis has shown that frailty was an independent predictor of clinical cirrhosis stage progression from mild to more advanced stages or death [19]. Therefore, therapeutic intervention aimed at the reversing frailty in patients with cirrhosis is needed.

In the present study, a 16-week enteral nutritional support with BCAA-enriched solution resulted in an improvement of frailty in frail compensated cirrhotic patients. To the best of our knowledge, this study is the first that specifically evaluates the addition of BCAA to the standard-of-care to correct frailty in patients with cirrhosis. We were able to demonstrate that a 16-week of twice daily BCAA supplementation resulted in a significant decrease in the LFI compared to the control group (-0.36 vs. -0.15, P < 0.001). Of note, approximate one-third of patients in the BCAA group had frailty resolution at the end of the trial compared with none in the control group. So far, frailty and sarcopenia, a condition of low muscle mass, quality, and strength, have been documented as an important issue in patients with cirrhosis [2, 21, 22]. The pathogenesis of sarcopenia is mainly caused by an imbalance between muscle breakdown and formation. A number of mechanisms have been proposed to be linked with a loss of muscle mass and strength in cirrhosis; for example, chronic inflammation causing an increase in pro-inflammatory cytokines, impaired nutrient intake and malabsorption, and hormonal changes-associated with chronic liver disease [21, 23]. In addition, cirrhosis is considered as a hypermetabolic state, which associated with an increased in fatty acid oxidation and gluconeogenesis resulting in low plasma BCAA levels. In the skeletal muscles, BCAA are the precursors for protein synthesis, and serve as the preferential energy source, therefore, low plasma BCAA levels may result in muscle degradation and impaired muscle strength. From the pathophysiologic standpoint, physical frailty and a loss of muscle strength are strongly related, for instance, the ‘grip strength’ and ‘timed chair-stands’ tests are the methods of measuring muscle strength. Therefore, we believe that the positive effect of oral BCAA in frailty improvement is by correcting low muscle strength accompanying chronic liver disease.

In the present study, an improvement of LFI at the end of follow-up was observed not only in the BCAA group but also in the control group. We proposed that an improvement of LFI in the control group could be attributed to the dietary and exercise advice provided during the run-in period. A recent placebo-controlled trial evaluating the role of 12-week BCAA supplementation in cirrhotic patients with sarcopenia has demonstrated that LFI significantly decreased in both the BCAA and control arms at the end of follow-up [15]. This finding was in line with the result of our study, however, the difference in the changes of LFI between two groups in the aforementioned study was not statistically significant. The discrepancy between this study and our could be explained by the fact that the study by Hernández-Conde et al. included a relatively lower proportion of frail patients (37.5%). Regarding changes in muscle mass, the delta SMI at week 16 was higher in the BCAA group; however, no statistical significance was found. This finding could be attributed to inadequate sample size to detect this difference. Nonetheless, compared with the baseline value, the BCAA group had a significant increase in SMI at the end of the follow-up. The benefit of BCAA supplementation in increasing muscle mass in patients with cirrhosis has been demonstrated in previous studies [16, 24, 25]. Hence, a recently published study failed to demonstrate an improvement in muscle mass after adding BCAA for 6 months in cirrhotic patients with sarcopenia. These conflicting results could be attributed to the difference in the preparation of oral BCAA products. In the study by Mohta et al., the form of BCAA was oral granules which mainly contains only BCAA, whereas our study and others used enteral nutrition BCAA preparation. Enteral BCAA formula is an BCAA-enriched complete elemental diet which contains protein, fat and carbohydrate providing approximately 200 kcal/serving. This finding supports the idea that cirrhosis is a hypermetabolic state, and most patients are in undernutrition state. Therefore, nutritional intervention in cirrhosis should emphasize on encouraging the patients to achieve the adequate energy and protein intake. In addition, several studies have demonstrated that the benefit of BCAA on skeletal muscle in cirrhosis was enhanced by adding exercise [26, 27]. A recent pilot study has shown that a 12-week exercise intervention was able to improve aerobic fitness as tested by 6-minute walk test in patients with cirrhosis [28]. In our study, all patients received exercise counseling to achieve a target of 150 min/week with a telephone visit every 2 weeks. Taken together, this information supports that adding exercise intervention to the standard-of-care in patients with cirrhosis is of particular importance.

Another relevant finding of our study is that, apart from the benefit on frailty and skeletal muscle mass, BCAA supplementation also resulted in an improvement in the QoL. Several studies have demonstrated that QoL was dramatically impaired in patients with cirrhosis, and a poor QoL was associated with an increased risk of unplanned hospitalization and short-term mortality [29, 30]. In addition, a recent prospective study has depicted that frailty was associated with a decrease in QoL independent of the cirrhosis severity [8]. In the present study, the BCAA group had a significant improvement in the physical component score of the SF-36 questionnaire as well as its 4 subscores. This favorable effect is likely to be related with an increase in muscle mass and strength after BCAA supplementation. Hence, a previous study failed to show the benefit of BCAA granules supplementation for 12 weeks on QoL in patients with cirrhosis [31]. This finding again supports the idea that the benefit of BCAA in cirrhotic patients partly depends on its composition and formula.

In the present study, the BCAA group had a significant improvement in serum albumin at week 16, however, there was no significant changes in the Child-Pugh, MELD and MELD-Na scores. This absence of efficacy of BCAA on the staging of cirrhosis could be explained by several reasons. First, our study had a relatively short treatment duration (16 weeks). Meanwhile, previous studies showing an improvement in Child-Pugh or MELD score in cirrhotic patients after BCAA treatment had at least 6 months follow-up period [14, 32]. Second, the majority of our patients were Child-Pugh A cirrhosis without previous history of hepatic decompensation. According to the natural history of compensated cirrhosis, clinical trials of intervention to improve cirrhosis severity scores in patients with mild liver impairment would require larger sample size.

We acknowledged several limitations in the present study. Firstly, this study was a single-center, open-label study. However, the majority of outcomes in this study were objective, and LFI was assessed by the investigators who were unaware of treatment group. These could reduce biases that may occur in an open-label trial. Secondly, we did not assess changes in muscle mass by computer tomography (CT) scan, which is currently the reference method for muscle mass evaluation in cirrhosis. Hence, a previous study has shown that low SMI as evaluated by BIA was associated with morbidity and mortality in patients with cirrhosis [33]. Moreover, previous studies has demonstrated a modest correlation between SMI as obtained by BIA and by CT scan, particularly in patient without ascites [34, 35]. Given that our patients were free of any hepatic decompensation for at least 6 months, we believe that the favorable effect of BCAA on muscle mass demonstrated in this study is clinically relevant. Thirdly, since the population of this study was compensated cirrhosis; therefore, our findings may not be applied to those in decompensated stage. Further studies evaluating the role BCAA in decompensated cirrhosis, particularly comparing BCAA with other late evening nutritional supplementations would be of special interest. Lastly, as the nutritional formula used in this study contains calories from carbohydrate, fat, and BCAA as a part of protein source, it is difficult to determine whether the positive effects of the intervention are attributable to BCAA supplementation or nutritional improvement. This issue deserves to be clarified in future studies.

Despite these limitations, our study provides new insights into the management of cirrhosis. The addition of BCAA for 16 weeks to the standard-of-care was able to improve physical frailty in frail compensated cirrhotic patients. In addition, this intervention also resulted in an increase in muscle mass and the physical component score of QoL in these patients. Future studies are needed in larger cohorts of patients, and to determine the optimal dosage and duration of BCAA supplementation.

## Data Availability

The datasets used and/or analyzed during the current study are available from the corresponding author on reasonable request.

## List of Abbreviations

MELD: Model for end-stage liver disease
QoL: Quality of life
BCAA: Branched-chain amino acids
LFI: Liver Frailty Index
PHT: Portal hypertension
GI: Gastrointestinal
HE: Hepatic encephalopathy
AKI: Acute kidney injury
HCC: Hepatocellular carcinoma
BUN: Blood urea nitrogen
BIA: Bioelectrical impedance analysis
SKI: Skeletal muscle index
HRQoL: Health-related quality of life
SD: Standard deviation
BMI: Body mass index
HBV: Hepatitis B virus
HCV: Hepatitis C virus
Hb: Hemoglobin
WBC: White blood cell
AST: Aspartate transaminase
ALT: Alanine transaminase
ALP: Alkaline phosphatase
aPTT: Activated partial thromboplastin time
PT: Prothrombin time
HGS: Hand grip strength
CST: Chair stands test
